# Draft genome sequence of *Coxiella burnetii* Dog Utad, a strain isolated from a dog-related outbreak of Q fever

**DOI:** 10.1002/nmi2.55

**Published:** 2014-06-26

**Authors:** F D'amato, M Million, S Edouard, J Delerce, C Robert, T Marrie, D Raoult

**Affiliations:** 1 Unité de Recherche sur les Maladies Infectieuses et Tropicales Emergentes, UM63, CNRS 7278, IRD 198, INSERM 1095, Aix-Marseille UniversitéMarseille, France; 2 Faculty of Medicine, Dalhousie UniversityHalifax, Nova Scotia, Canada

## Abstract

*Coxiella burnetii* Dog Utad, with a 2 008 938 bp genome is a strain isolated from a parturient dog responsible for a human familial outbreak of acute Q fever in Nova Scotia, Canada. Its genotype, determined by multispacer typing, is 21; the only one found in Canada that includes Q212, which causes endocarditis. Only 107 single nucleotide polymorphisms and 16 INDELs differed from Q212, suggesting a recent clonal radiation.

*Coxiella burnetii* is a Gram-negative bacterium with a complex intracellular cycle belonging to the γ-proteobacteria 1. To date, 13 genomes are available from the NCBI 2. Our strain, Dog Utad, was isolated in Marseille from a dog responsible for a Q fever familial outbreak in Nova Scotia, Canada 3. This female dog was a hound that had caught rabbits during its pregnancy and gave birth to four pups. All of them died within 24 h after birth. All three family members (mother, father and son) who were present during the delivery, developed pneumonia in the 2 weeks following parturition. Index case was the mother, who helped with the delivery and cleaned up afterward. Serological data confirmed acute Q fever in the mother and the son and *C. burnetii* was isolated by shell vial technique 4 from the dog uterus, which was removed 70 days after parturition, frozen and shipped to our laboratory 5.

Genotyping was performed on the strain using multi-spacer sequence typing (MST) 6, a technique based on the variability of ten intergenic sequences. The genotype MST 21 was identified and confirmed by BLAST of the genome 7 against Cox sequences (10). We compared the Dog Utad strain to the seven available strains. A comparison of the COG categories showed that Dog Utad follows the same trend as the other available *C. burnetii* genomes, but with more similarities with CbuG_Q212, a genome previously deposited in GenBank corresponding to a strain from a Canadian man presenting with Q fever endocarditis and having the same genotype (MST21).

*Coxiella burnetii* Dog Utad reads best covered (98%) the *Coxiella burnetii* CbuG_Q212 genome with a maximum coverage of 1482 and an average coverage of 173. Moreover, the final Dog Utad sequence was the same size as CbuG_Q212 (2 Mb) and there were only 80 gaps (for a total of 52 047 bp). The genome is characterized by a consensus sequence of 2 008 938 bp (G+C content 44%). It encodes 1896 proteins and carries 44 tRNA and one ribosomal operon.

To the best of our knowledge after analysing 335 strains with available MST from around the world, MST21 was the only genotype identified in Canada. Two other strains from humans presenting with Q fever endocarditis, two from cats and one other from a dog from Canada were also identified as MST21. The uniformity of geographical and genotypic criteria allows us to define the Canadian geotype, which corresponds to the genotype MST21, infects cats, dogs and humans and is responsible for acute Q fever and endocarditis in the Canadian population. It was also found in two French patients 6 and in Alberta, Canada.

We found only 123 mutations (70 of them in putative open reading frames (ORF)) when compared with CbuG_Q212: 107 single nucleotide polymorphisms (SNPs; 67 in ORF), eight insertions (one ORF) and eight deletions (two in ORF). Forty-seven of the 70 are non-synonymous mutations, corresponding to 44 mutated genes. These genes encode for 17 proteins involved in metabolism, five transporters, three membrane proteins, three proteins of signal transduction, three translation proteins, two transcription proteins, two chaperone proteins, three hypothetical proteins, one type IV secretion system protein, one DNA replication protein, one protein involved in cellular processes, one organic solvent tolerance protein, one isomerase and one stress protein (Table1). This very low number of SNPs suggests a very short genetic distance between these two genomes, suggesting a recent clonal radiation of *C. burnetii* MST21 in Canada.

**Table 1:** Non-synonymous point mutations in *Coxiella burnetii* Dog Utad compared with *Coxiella burnetii* Q212

Position^a^	Type	Gene ID	Annotation	Nucleotide change (Q212→Dog Utad)	Amino acid change (Q212→Dog Utad)
26219	SNP	CbuG_0030	Hypothetical protein	C→A	Ala→Glu
57338	SNP	CbuG_0066	Thioredoxin peroxidase	A→C	Ser→Ala
77850	SNP	CbuG_0091	Transporter-sodium dependent	A→C	Thr→Pro
100854	SNP	CbuG_0107	Biotin carboxylase	G→C	Gly→Ala
329943	SNP	CbuG_0357	Ribosomal protein α-l glutamate ligase	G→A	Gly→Glu
366913	SNP	CbuG_0400	IcmJ	G→A	Glu→Lys
366926	SNP	CbuG_0400	IcmJ	A→G	Glu→Gly
393251	SNP	CbuG_0426	Sodium/proton antiporter protein	T→C	DEL20aa
503017	SNP	CbuG_0540	Exported membrane spanning protein	T→A	Phe→Tyr
504897	SNP	CbuG_0540	Exported membrane spanning protein	A→G	Thr→Ala
543181	SNP	CbuG_0577	Bacterial protein translation initiation factor 2	G→A	Val→Met
610658	SNP	CbuG_0645	Outer membrane lipoprotein	C→T	Met→Leu
719445	SNP	CbuG_0753	Na^+^/H^+^ antiporter	T→C	Gln→Arg
736723	SNP	CbuG_0769	Malate dehydrogenase	G→A	Arg→Gln
763310	SNP	CbuG_0791	Phosphoglycolate phosphatase	T→C	Phe→Ser
803836	SNP	CbuG_0828	Glycine-rich RNA-binding protein	T→C	Met→Leu
836313	SNP	CbuG_0860	Transcription-repair coupling factor	G→T	Ser→Tyr
894420	SNP	CbuG_0919	Two component system histidine kinase	T→G	Ile→Ser
914733	SNP	CbuG_0941	ABC transporter	T→G	Leu→Phe
928574	SNP	CbuG_0953	Aspartokinase	C→T	Pro→Leu
974371	SNP	CbuG_1002	Carboxylesterase	A→T	Glu→Asp
1006992	SNP	CbuG_1039	Cytochrome d ubiquinol oxidase subunit I	T→C	Ser→Gly
1050734	SNP	CbuG_1088	Endonuclease/exonuclease/phosphatase family protein	C→G	Ala→Pro
1052481	SNP	CbuG_1090	UDP-*N*-acetyl glucosamine 6 dehydrogenase	C→T	Ala→Val
1065901	SNP	CbuG_1106	Colicin V production protein	A→C	Leu→Trp
1076084	SNP	CbuG_1117	Aminoglycoside *N*-6′ acetyltransferase	G→A	Ala→Val
1164183	SNP	CbuG_1199	Ribosomal-protein-S18-alanine acetyltransferase	T→C	Asn→Asp
1190949	SNP	CbuG_1221	Response regulator	A→C	Val→Gly
1292064	SNP	CbuG_1326	NAD-dependent epimerase/dehydratase family	G→T	Thr→Lys
1314867	SNP	CbuG_1357	GTP cyclohydrolase II	T→G	Glu→Ala
1549656	SNP	CbuG_1595	ATP-dependent DNA helicase Rec G	G→T	Ser→Arg
1615472	SNP	CbuG_1668	Membrane alanine aminopeptidase	A→G	Leu→Pro
1662811	SNP	CbuG_1713	Hypothetical protein	T→C	Phe→Xaa
1790891	SNP	CbuG_1860	Type 4 pili biogenesis protein (nucleotide-binding protein)	G→A	Glu→Lys
1802885	SNP	CbuG_1874	UDP-3-*O*-[3-hydroxymyristoyl] *N*-acetylglucosamine deacetylase	C→T	Gly→Asp
1807487	SNP	CbuG_1879	UDP-*N*-acetylenolpyruvoylglucosamine reductase	T→G	Asn→His
1899207	SNP	CbuG_1975	Multidrug resistance protein D	G→T	Gly→Cys
1902387	SNP	CbuG_1983	Carboxylesterase	T→G	Ser→Ala
1904423	SNP	CbuG_1985	Organic solvent tolerance protein	A→G	Tyr→Cys
1906869	SNP	CbuG_1985	Organic solvent tolerance protein	A→C	Glu→Asp
1907022	SNP	CbuG_1987	Peptidyl-prolyl *cis*-*trans* isomerase	G→A	Val→Ile
1910350	SNP	CbuG_1990	Universal stress protein A	G→A	Ala→Thr
1933346	SNP	CbuG_2020	ATP-dependent endopeptidase hsl proteolytic subunit	C→G	Phe→Leu
1934809	SNP	CbuG_2021	ATP-dependent endopeptidase hsl ATP-binding subunit	T→C	Met→Thr
1185060	Insertion	CbuG_1215	CoA-transferase family III protein	–TGG	INS His
479626	Deletion	CbuG_0513	NADH quinone oxidoreductase subunit L	G–	DEL5aa
491265	Deletion	CbuG_0526	Hypothetical protein	T–	DEL1aa

Single nucleotide polymorphisms and INDELs after comparison between *Coxiella burnetii* Dog_Utad and *Coxiella burnetii* Cbu_Q212.

a Position in *C. burnetii* Cbu_Q212.

## Nucleotide Sequence Accession Numbers

Strain Dog Utad has been deposited in GenBank under the project accession number PRJEB4294. The version described in this article is the first version, PRJEB4294.
